# Comparison of Selection Signatures between Korean Native and Commercial Chickens Using 600K SNP Array Data

**DOI:** 10.3390/genes12060824

**Published:** 2021-05-27

**Authors:** Sunghyun Cho, Prabuddha Manjula, Minjun Kim, Eunjin Cho, Dooho Lee, Seung Hwan Lee, Jun Heon Lee, Dongwon Seo

**Affiliations:** 1 Division of Animal and Dairy Science, Chungnam National University, Daejeon 34134, Korea; cshcshh@cnu.ac.kr (S.C.); prabuddhamanjula@yahoo.com (P.M.); mjkim6023@naver.com (M.K.); flunecmer@naver.com (E.C.); stonecold@daum.net (D.L.); slee46@cnu.ac.kr (S.H.L.); junheon@cnu.ac.kr (J.H.L.); 2 Bio-AI Convergence Research Center, Chungnam National University, Daejeon 34134, Korea

**Keywords:** Korean native chicken, signature of selection, quantitative trait locus

## Abstract

Korean native chickens (KNCs) comprise an indigenous chicken breed of South Korea that was restored through a government project in the 1990s. The KNC population has not been developed well and has mostly been used to maintain purebred populations in the government research institution. We investigated the genetic features of the KNC population in a selection signal study for the efficient improvement of this breed. We used 600K single nucleotide polymorphism data sampled from 191 KNCs (NG, 38; NL, 29; NR, 52; NW, 39; and NY, 33) and 54 commercial chickens (Hy-line Brown, 10; Lohmann Brown, 10; Arbor Acres, 10; Cobb, 12; and Ross, 12). Haplotype phasing was performed using EAGLE software as the initial step for the primary data analysis. Pre-processed data were analyzed to detect selection signals using the ‘rehh’ package in R software. A few common signatures of selection were identified in KNCs. Most quantitative trait locus regions identified as candidate regions were associated with traits related to reproductive organs, eggshell characteristics, immunity, and organ development. Block patterns with high linkage disequilibrium values were observed for *LPP*, *IGF11*, *LMNB2*, *ERBB4*, *GABRB2*, *NTM*, *APOO*, *PLOA1*, *CNTN1*, *NTSR1*, *DEF3*, *CELF1*, and *MEF2D* genes, among regions with confirmed selection signals. NL and NW lines contained a considerable number of selective sweep regions related to broilers and layers, respectively. We recommend focusing on improving the egg and meat traits of KNC NL and NW lines, respectively, while improving multiple traits for the other lines.

## 1. Introduction

‘Selection’ refers to a phenomenon in which genetic and phenotypic characteristics are fixed within a population. Continuous positive selection, either natural or artificial, affects allele diversity. During this process, a specific allele can be fixed in a specific population, and adjacent alleles are gradually fixed together. This results in an increase or decrease in the genotype frequencies of a particular gene and is commonly referred to as ‘selective sweep’ [1,2]. In an analysis of selection signals, traces of selective sweep are examined within genes. Therefore, the study of selection signals can be used to identify a genomic region fixed within a population undergoing selection, or to search for genes or genomic regions associated with a specific trait through comparative analysis. The genome-wide association study approach is the most representative method used to analyze the association between phenotype and genotype. However, in a genome-wide association study, information regarding phenotype measurements is necessary. The results can be affected by errors resulting from incorrect phenotype information and influenced by a biased genotype distribution. However, the analysis of selection signals is free from some limitations associated with other methods. Therefore, it is considered an appropriate analytical method for fixed unknown traits or gene-tracking studies in populations without quantitative phenotype information. There are many selection-signal analysis methods, but single allele frequencies or haplotype information have recently been used to detect selective sweep. Among the available methods, the approach based on extended haplotype homozygosity (EHH) uses haplotype and long-range linkage disequilibrium (LD) information and is reportedly able to select significant high-homozygosity regions with greater accuracy, compared with a single allele frequency approach [3,4,5].

The chicken is an important livestock species and protein source for humans. Additionally, chickens are amenable to improvement because they have a short life cycle and can produce a large offspring population, compared with other livestock animals. Commercially, improvements can be made for layer (producing eggs) or broiler (producing meat) chickens. With respect to broilers, from 1957 to 2005, their growth rate increased by more than 400%, while the feed conversion ratio (FCR) decreased by 50% [6].

The Korean native chicken (KNC) nearly became extinct because of the Korean War and industrialization, but in 1992, five pure lines were restored through a restoration project implemented by the government. These lines are categorized as follows, according to the colors of their feathers: red–brown (NR), yellow–brown (NY), grey–brown (NG), black (NL), and white (NW). Until recently, these KNC lines have been maintained for preservation purposes, and have not been improved. Additionally, there is little information available regarding the improvement of economic traits. The KNC reportedly has a lower growth rate and feed efficiency than broiler chickens [7,8]. For these reasons, the production of KNCs in Korea is only 3% of the total broiler production (Animal and Plant Quarantine Agency, 2018). Therefore, the profitability of KNC production must be improved by applying selective breeding that considers the genetic characteristics of economic traits for each chicken line. Furthermore, potential areas of improvement should be identified, such as whether the genetic characteristics of each KNC line are related to meat or egg traits. Therefore, this study was performed to identify genetic similarities and differences among the lines by comparing selection signatures of broiler and layer populations with the five KNC lines.

## 2. Materials and Methods

### 2.1. Sample Genotyping and Genetic Diversity Analysis

In total, 245 samples comprising purebred KNCs and commercial broilers and layers were analyzed in this study. The KNC group was collected from the Korean National Institute of Animal Science in 2011 and it was further divided into five lines (NG, 38; NL, 29; NR, 52; NW, 39; and NY, 33). Broilers and layers were obtained from commercial farms in 2017 and these were divided into three (Arbor Acres [Ab], 10; Cobb [CB], 12; and Ross [RS], 12) and two (Hy-line Brown (HL), 10; and Lohmann Brown (LO), 10) varieties, respectively. Genomic DNA (gDNA) was extracted from blood or tissue samples from all birds using PrimePrep™ Genomic DNA Isolation kits (GeNetBio, Daejeon, Korea). The concentration and purity of extracted gDNA were investigated using a NanoDrop spectrophotometer (Thermo Fisher Scientific, Waltham, MA, USA), and the gDNA was stored at −20 °C until use.

With these gDNA samples, data regarding 547,784 single nucleotide polymorphisms (SNPs) were obtained using a 600K Affymetrix Axiom^®^ array (Affymetrix, Santa Clara, CA, USA). For quality control, SNPs with a minor allele frequency of ≥5% and a call rate of 90% were selected for further analysis. Eventually, 540,560 SNPs distributed across Gallus gallus chromosomes 1–28 were used in this study.

Three general diversity analyses were performed to assess the genetic diversity of the entire population. First, principal component analysis was performed using PLINK1.9 software [9]. The results were confirmed in scatter plots based on the four principal component (PC) axes with the best explanatory power according to their total loading values. Second, population admixture analysis was performed using ADMIXTURE 1.3 software [10]. The number of common ancestors (*K*-value) was calculated from a range of 2–15, and the result was confirmed using a bar plot. Third, a phylogenetic tree based on Nei’s genetic distance was constructed using the ‘poppr’ package in R software [11].

### 2.2. LD Analysis and the Detection of Regions Exhibiting Selection Signatures Based on iHS and Rsb between Populations

To detect selection signals, phasing was performed using genotype data for each chromosome in Eagle v2.4.1 software [12]. Haplotype phasing data were used to calculate the integrated haplotype homozygosity score (iHS) and extended haplotype homozygosity ratio between populations (Rsb) with the ‘rehh’ package in R software (Equation (1)) [13]. Candidate regions were determined using the sliding window method. Each window had a size of 25 kb, with an overlap of 12.5 kb. When more than four significant SNPs (*p* < 0.01) were included in a window, it was considered a candidate region.

(1) $$EHHS_{s,t}=\frac{1}{n_{s}\left(n_{s}-1\right)}\left(\overset{K_{s,t}}{\underset{k=1}{\sum }}n_{allele1}\left(n_{allele1}-1\right)+\overset{K_{s,t}}{\underset{k=1}{\sum }}n_{allele2}\left(n_{allele2}-1\right)\right)\;uniHS=ln\left(\frac{iHH^{allele1}}{iHH^{allele2}}\right),\;\;iHS=\frac{uniHS-mean\left(uniHS\right)}{sd\left(uniHS\right)}\;h_{s}=\frac{n_{s}}{1-n_{s}}\left(1-\frac{1}{n_{s}^{2}}\left(n_{allele1}^{2}+n_{allele2}^{2}\right)\right)\;nEHHS_{s,t}=\frac{1-h_{s,t}}{1-h_{s}}\;unRsb=ln\left(\frac{inES_{pop1}}{inES_{pop2}}\right),\;\;Rsb=\frac{unRsb-median\left(unRsb\right)}{sd\left(unRsb\right)}$$

$n_{s}$: Total number of haplotype;
$n_{allele1}$: Total number of haplotype for allele1;
$n_{allele2}$: Total number of haplotype for allele2;
$inES_{pop1}$: integrated haplotype homozygosity score (*nEHHS*) for pop1;
$inES_{pop2}$: integrated haplotype homozygosity score (*nEHHS*) for pop2.

A heatmap plot was created by calculating the linkage coefficient of correlation (*r^2^*) between SNPs included in a candidate region using the ‘LDheatmap’ package in R software [14]. Additionally, an LD block was defined as the area where more than four consecutive SNPs with *r^2^* ≥ 0.6 were located.

### 2.3. Gene and Quantitative Trait Locus (QTL) Annotation

Annotation was performed to check the genetic information associated with each candidate selection signal region. Genetic information was acquired from corresponding entries in the Ensembl database (Gallus_gallus-5.0) using the ‘biomaRt’ package in R software [15]. QTL annotation was performed to check the QTL information associated with each candidate region. Gallus_gallus-5.0 QTL information was extracted from the Animal QTL Database (https://www.animalgenome.org/cgi-bin/QTLdb/GG/index (accessed on 20 January 2021)), and candidate selection regions were identified.

## 3. Results

### 3.1. General Diversity and Sliding Window Analyses

**Figure 1:**
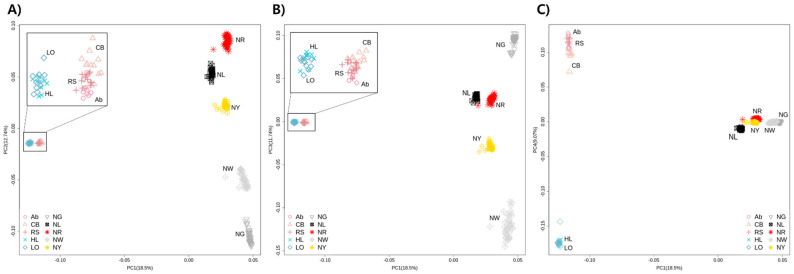
Principal component analysis results for commercial (broilers and layers) chicken breeds (Ab, Arbor Acre broiler; CB, Cobb broiler; RS, Ross broiler; HL, Hy-line Brown layer; and LO, Lohmann Brown layer) and five Korean native chicken (KNC) lines (NG, grey–brown; NL, black; NR, red–brown; NW, white; and NY, yellow–brown). (**A**) Plot with principal component (PC) 1 and PC2. (**B**) Plot with PC1 and PC3. (**C**) Plot with PC1 and PC4.

**Figure 2:**
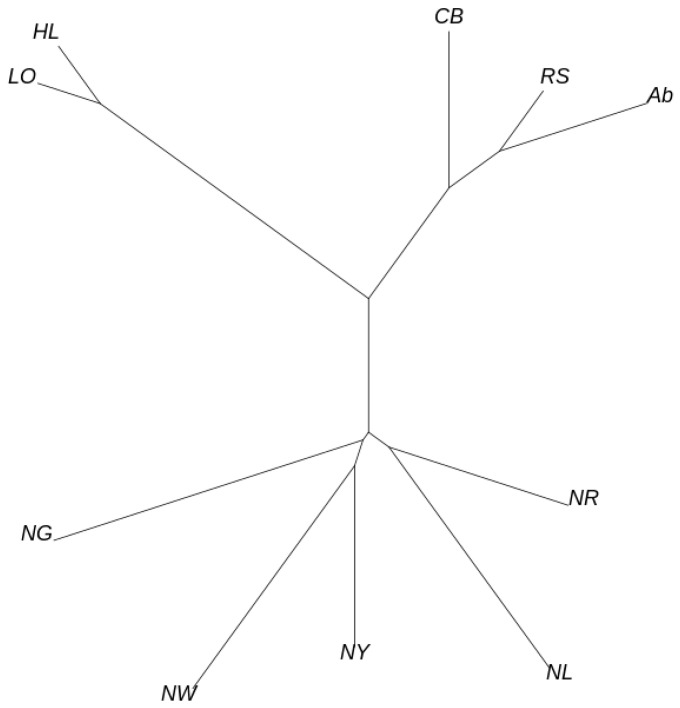
Phylogenetic tree including commercial (broilers and layers) chicken breeds (Ab, Arbor Acre broiler; CB, Cobb broiler; RS, Ross broiler; HL, Hy-line Brown layer; and LO, Lohmann Brown layer) and five KNC lines (NG, grey–brown; NL, black; NR, red–brown; NW, white; and NY, yellow–brown).

**Figure 3:**
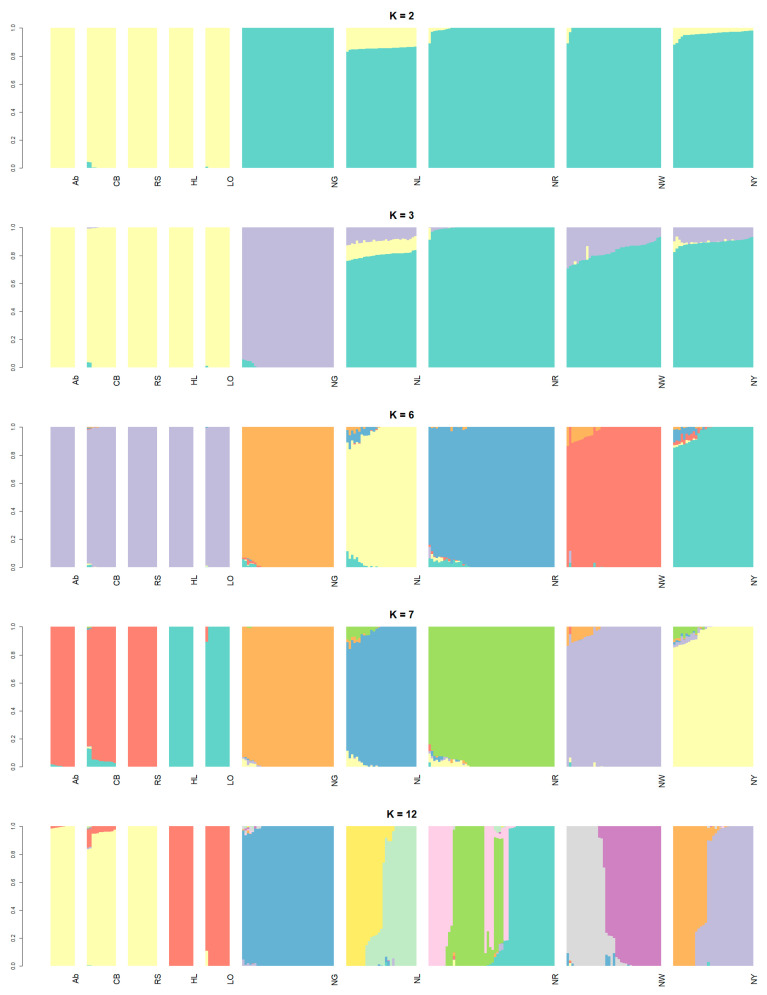
Results of admixture analysis of commercial (broilers and layers) chicken breeds (Ab, Arbor Acre broiler; CB, Cobb broiler; RS, Ross broiler; HL, Hy-line Brown layer; and LO, Lohmann Brown layer) and five KNC lines (NG, grey–brown; NL, black; NR, red–brown; NW, white; and NY, yellow–brown).

Principal component analysis was performed to confirm genetic similarities and differences among the chicken populations sampled in this study (Figure 1). PC1–4 explained 18.50%, 12.74%, 11.74%, and 9.07% of the total variance, respectively, and thus had substantial explanatory power. All KNC lines (NG, NL, NR, NW, and NY) were clustered separately. The commercial chicken breeds were clustered into broiler (Ab, CB, and RS) and layer (HL and LO) groups. In particular, the KNC lines and commercial chicken breeds could be distinguished based on PC1, which had the greatest explanatory power. The commercial chicken breeds also clustered separately from the KNC lines in the phylogenetic tree constructed based on Nei’s genetic distance, forming their own subtree (Figure 2). Furthermore, the NL and NR lines were located in their own subtree, as were the NW and NY lines. The outcomes of admixture analysis confirmed the previous results in greater detail (Figure 3 and Figure S1). With two common ancestors (*K* = 2), there was a clear separation between the commercial and native chicken breeds. The largest difference between the NG and NR lines was observed when *K* = 3. Additionally, excluding NG, the remaining KNC lines shared at least 70.88% of their genetic makeup with the NR line, consistent with the phylogenetic analysis results. When *K* = 6, the five KNC lines were completely separated, and when *K* = 7, broilers and layers were separated. At the optimal genetic component value, *K* = 12, KNC lines were separated from the broilers and layers. In particular, mixing of genetic components was confirmed in all lines except NG, and more than 99.89% of the single components were identified. To determine the regions with selection signatures, the entire genome was divided into 25-kb windows (each with an overlap of 12.5 kb). In total, 37,238 windows were identified; the mean number of SNPs per window was 14.17098.

### 3.2. Detection of Selection Signatures and QTL Annotation

**Figure 4:**
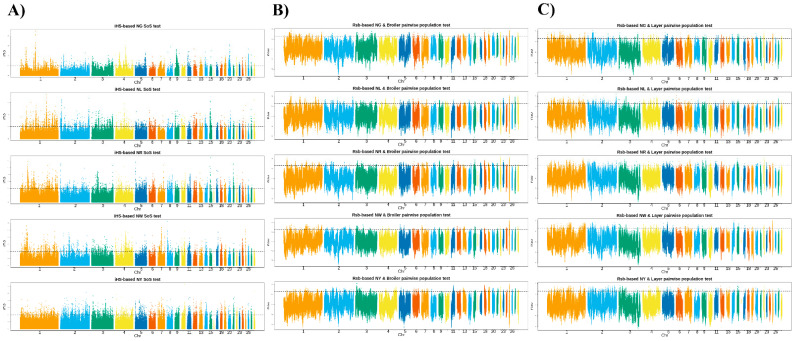
Results of comparative selection signal analysis between commercial chickens (broilers and layers) and KNCs based on the extended haplotype homozygosity ratio between populations (Rsb) and the integrated haplotype homozygosity score (iHS). (**A**) Regions in the KNC population exhibiting selection signals based on the iHS. (**B**) Pairwise Rsb indicating selection signals in the KNC population vs. the broiler population. (**C**) Pairwise Rsb indicating selection signals in the KNC population vs. the layer population. Black lines indicate threshold at *p* < 0.01.

From the haplotype-based iHS and Rsb analyses, within- and between-group selection signals for the five KNC lines were identified. Some overlaps were found, but in most instances, selection signals were detected in distinct areas for each line (Figure 4).

In terms of within-population selection signals in the KNC lines, 125, 168, 135, 114, and 98 signals were identified in the NG, NL, NR, NW, and NY lines, respectively (*p* < 0.01). iHS analysis could not identify common selection signal areas in the KNC lines. 

However, based on QTL annotation, significant effects were found for features related to immunity (spleen weight, fowl typhoid susceptibility, Marek’s disease virus antibody titer, infectious bronchitis virus antibody titer, and alternative complement activation by red blood cells) and eggshell traits. Layer-specific selection signals were identified in QTL regions related to eggshell traits (strength and thickness), production (feed conversion ratio, FCR), and fear-associated behaviours (e.g., feather pecking and aggressive behaviours) (Table S1).

Upon comparing selection signals with the broiler population, 66, 65, 80, 52, and 46 areas with selection signals were identified in the NG (Rsb ≥ 2.549), NL (Rsb ≥ 2.557), NR (Rsb ≥ 2.556), NW (Rsb ≥ 2.544), and NY (Rsb ≥ 2.550) lines, respectively. QTL annotation revealed significant QTL regions related to long-term development (ileum length, ileum weight, and duodenum length), reproduction (ovary percentage and ovary weight), production (FCR and feed intake), fear—tonic immobility duration (in most KNC lines), exterior traits (comb weight and wattle weight), and other traits (Table S2).

Upon comparing selection signals with the layer population, 134, 113, 132, 111, and 122 areas with selection signals were identified in the NG (Rsb ≥ 2.578), NL (Rsb ≥ 2.569), NR (Rsb ≥ 2.564), NW (Rsb ≥ 2.570), and NY (Rsb ≥ 2.562) lines, respectively. QTL annotation revealed significant QTL domains in most of the KNC lines related to long-term development (ileum weight, gizzard weight, and heart weight), reproduction (egg number, ovary percentage, and ovary weight), eggshell traits (color and strength), production (dry matter intake, FCR, and feed intake), fear—tonic immobility duration, feather pecking, and exterior traits (comb weight and wattle weight) (Table S3).

### 3.3. LD Block Profiling for Significant Selection Signal Regions

Different LD block patterns were found for each line in the selection signal analysis. In this study, an LD block refers to an instance of more than four consecutive SNPs with an LD coefficient (*r^2^*) ≥ 0.6. For the KNCs, 18,008, 16,134, 19,827, 19,006, and 16,605 LD blocks were identified in the NG, NL, NR, NW, and NY lines, respectively. For the commercial chicken breeds, 13,448 and 14,540 blocks were identified in the broilers and layers, respectively, which were small numbers compared with the numbers of blocks in the KNCs. Among the LD blocks identified throughout the genome, candidate genes with greater selection pressure were extracted and visualised in an LD heatmap. In particular, block patterns with high LD coefficients were observed for the *LPP*, *IGF11*, *LMNB2*, *ERBB4*, *GABRB2*, *NTM*, *APOO*, *PLOA1*, *CNTN1*, *NTSR1*, *DEF3*, *CELF1*, and *MEF2D* genes.

## 4. Discussion

Although there are many differences between KNCs and commercial chicken breeds, the greatest difference is related to artificial selection. Traditionally, chickens were reared in the backyard to provide small amounts of eggs and meat for the household, not for economic purposes. Furthermore, the KNC population was restored to avoid extinction, not for economic benefits. Therefore, traces of positive selection for adaptation to the backyard environment in the wild-type chicken should be retained in the current KNC population. The purpose of this study was to conduct a basic diversity analysis of 191 KNCs and 54 commercial chickens using 600K SNP data, and to identify signals of positive selective sweep in KNCs that can be used for breed improvement.

Similar patterns were observed in both principal component and phylogenetic analyses. In the principal component analysis plots, broilers, layers, and the KNC lines clustered mainly into different groups, and similar trends were observed in subtrees on phylogenetic analysis. In particular, the KNC lines differed markedly from the commercial chickens, with large differences also evident among lines. Therefore, we performed three selection signal analyses for each line, comparing the lines to broilers and layers in iHS and Rsb. Genetic information for candidate regions exhibiting selection signals was extracted based on the locations of regions in the Gallus_gallus-5.0 genome (Table 1). However, because most candidate genes were unknown genes, QTL information was extracted regarding each putative selection signal region. Only a few candidate genes were shared among lines, according to the analyses. This is potentially because of genetic differences among KNC lines, as demonstrated by the diversity analysis. The results indicated that most QTL regions identified as candidate selection signal regions were related to biological characteristics such as reproductive organs, eggshell traits, immunity, and organ development.

**Figure 5:**
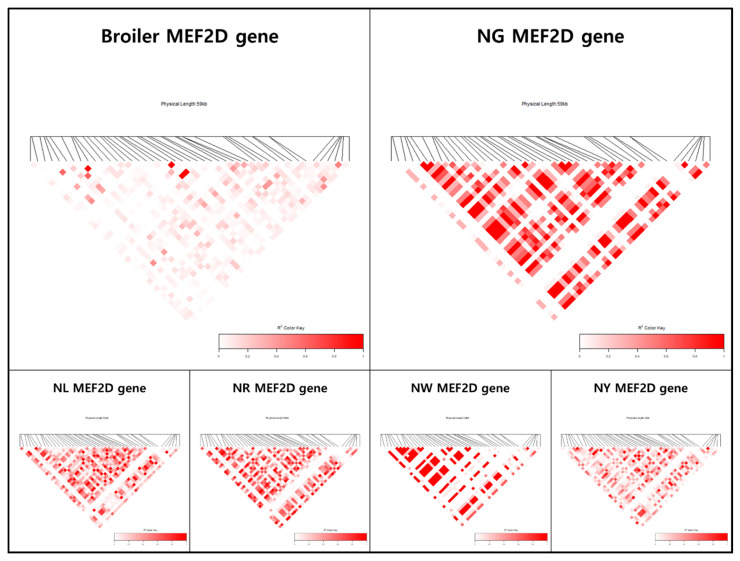
Heatmap of linkage disequilibrium blocks including the *myocyte-specific enhancer-binding factor 2D* (*MEF2D*) gene (Chr 25: 1,557,867–1,620,174 bp) in broilers and five KNC lines.

Backyard-reared chickens are able to move about freely, in contrast to cage-reared chickens. As KNCs can move continuously, differences related to motor abilities such as myocardial and muscle development are evident between these chickens and commercially bred chickens. In this study, candidate genes with these functions likely underwent selection (Figure S2). The *double PHD fingers 3* (*DPF3*) gene was shown to function in heart development, through a genome-wide association study comparing congenitally malformed hearts exhibiting tetralogy of Fallot and normal hearts [16]. The *DPF3* gene was specifically expressed during heart development in mice, chickens, and zebrafish, and the knockdown of *DPF3* in zebrafish resulted in myocardial contractility and incomplete heart formation; thus, *DPF3* was categorized as a major gene for heart development [17]. The *CUGBP Elav-like family member 1* (*CELF1*) gene encodes an RNA-binding protein that greatly affects the heart and skeletal muscles during early human embryonic development by regulating pre-mRNA alternative splicing, deadenylation, and mRNA decay and translation [18]. The knockdown of the *CELF1* gene in cardiomyocytes in chicken embryos confirmed that it is a major regulator of cardiomyocyte gene expression [19,20,21]. The *myocyte-specific enhancer-binding factor 2D* (*MEF2D*) gene is a member of the MEF2 family and acts as a major regulator in the production of various muscles [22,23,24]. Ouyang et al. (2020) [25] reported that the *MEF2D* gene generates four transcripts (*MEF2D-V1, MEF2D-V2, MEF2D-V3*, and *MEF2D-V4*), based on the results of tissue-specific transcriptome analysis at the embryonic stage in chickens. Among the four transcripts, *MEF2D-V4* was significantly associated with the embryonic phenotype and was highly expressed in embryonic leg muscles. This study confirmed the association of a selection signal with the *MEF2D* gene (Chr 25: 1,557,867–1,620,174 bp) in all KNC lines, in which a strong LD block was present compared with the broiler population (Figure 5).

KNCs have largely been raised as backyard chickens; they are thus more resistant to diseases and viruses, compared with commercial chickens, and likely consume coarser feed. To adapt to these conditions, a selective sweep might have occurred across the QTL regions related to immune responses, disease sensitivity, and long-term development (Figure S3). Among the candidate selection signal genes, the LIM domain containing the *preferred translocation partner in lipoma* (*LPP*), *immunoglobulin superfamily member 11* (*IGSF11*), and *lamin B2* (*LMNB2*) genes has been associated with immune responses and disease sensitivity. The *LPP* gene encodes a zyxin-related cell adhesion protein that regulates cytoskeletal tissue and cell migration and has been associated with some immune responses [26]. According to Feng et al. (2019) [27], SNPs located at the 3′-untranslated region of the *LPP* gene in humans have been associated with immunoglobulin A nephropathy. Liu et al. (2020) [28] confirmed that sensitivity to Salmonella enteritidis is associated with the genotypes of SNPs located in the *LPP* gene in chickens. The *IGSF11* gene is a member of the immunoglobulin superfamily and is mainly expressed in the brain and testes [29]. Zhang et al. (2019) [30] reported significant differences in *IGSF11* expression levels in the spleen and bursa between chickens with normal and low immunity, when the low immunity was induced by high-temperature stress. This indicates that *IGSF11* is associated with immune responses. Previous research revealed that the *LMNB2* gene was upregulated in the spleens of chickens infected with the reticuloendotheliosis virus and Marek’s disease virus, implying an association with immune responses [31,32]. These selective sweeps in KNCs are presumably adaptations to rearing under non-standardized backyard conditions. Based on findings in previous studies, we presume that KNCs have stronger immune responses because they exhibit higher genetic diversity, compared with commercial chickens, in the major histocompatibility complex B region, which plays major roles in adaptive and innate immune responses [33,34].

Outdoor-reared KNCs are more closely related to wild chickens due to a comparative lack of selective breeding compared with commercially reared chickens, which have undergone such selection for a long time. Generally, domesticated chickens exhibit reduced levels of aggression and fear [35,36]. Therefore, KNCs can respond more sensitively to external factors for survival purposes, compared with commercial chickens. Among the candidate genes detected in our analysis, we identified selection signals in the *Erb-b2 receptor tyrosine kinase 4* (*ERBB4*) and *γ-aminobutyric acid type A receptor β 2 subunit* (*GABRB2*) genes. *ERBB4* is a major gene involved in fear responses [37], and the *GABRB2* gene is reportedly associated with behavioral responses to anxiety in chickens and mice [38,39] (Figure S4).

Some of the genes exhibiting signals of selective sweep have functions related to reproductive organs (Figure S5). The *neurotrimin* (*NTM*) gene promotes outgrowth in resident sensory nerves in response to estrogen [40] and is associated with the regulation of oviduct development and differentiation in chickens, thus confirming a significant association with traits related to age at first egg [41,42]. The *contactin 1* (*CNTN1*) gene encodes an immunoglobulin family of cell adhesion molecules that contribute to the formation of connections among axons during the nervous system development [43]. Although the exact mechanism has not been confirmed, the *CNTN1* gene was reportedly differentially expressed (according to RNA sequencing analysis) in the ovaries and fallopian tubes (including the magnum, isthmus, and uterus) of chickens during egg production [44], and it has been identified as one of 25 candidate genes associated with an enhanced spawning ability [45]. *Neurotensin receptor 1* (*NTSR1*) is a receptor that acts in various ways in the central nervous system [46]. In particular, the *NTSR1* gene affects the anorexic pathway in chickens [47] and was identified as a major differentially expressed gene in a comparative ovarian transcriptome analysis of chickens exhibiting high and low levels of spawning [48]. The *DNA polymerase α 1, catalytic subunit* (*POLA1*) gene and the fatty acid and lipid metabolism-related *apolipoprotein O* (*APOO*) gene were identified as candidate genes that were strongly associated with weekly egg number [49]. The selective sweep of genes affecting the ovaries and fallopian tubes was confirmed in the KNC population, and this process presumably affected egg-laying and eggshell traits.

**Figure 6:**
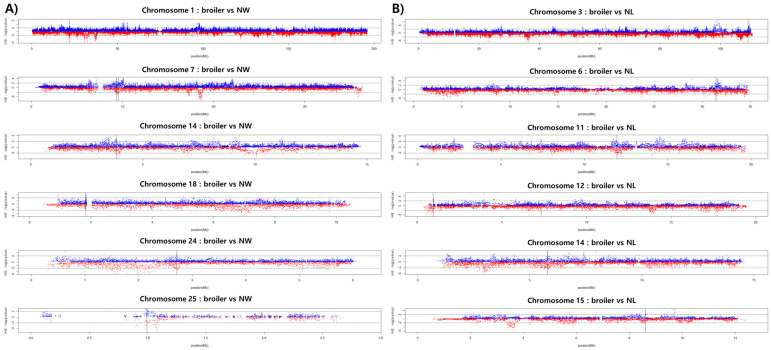
Selective sweep regions shared between commercial chickens and KNCs. (**A**) Commercial broilers and the KNC NW line. (**B**) Commercial layers and the KNC NL line. Black lines indicate shared selective sweep regions.

In this study, information regarding the selection signal regions and candidate selection signal genes in KNCs was elucidated. As genes exhibiting selection signals are related to immunity, fear behavioral responses, and myocardial development, positive selection might have been driven by the need to survive in a backyard environment. Although the selected genes are less related to economic traits, KNC traits can be used to maintain the robustness and environmental adaptability of commercially bred chickens. In addition, we indirectly confirmed the direction of improvement for each trait by comparing the within-group selection signals (iHS) between commercial chicken and KNC populations. Although phenotypic data were not included in our analysis, information regarding candidate selection signal genes and QTLs was extracted from the selective sweep regions shared between the commercial chicken and KNC populations (Table 2). Some notable results were observed. In the KNC NL line, shared selective sweep regions were not detected upon comparison with broiler chickens. However, a large number of shared regions were detected upon comparison with layer chickens. In contrast, shared selective sweep regions were detected between NW and broiler chickens, but not between NW and layer chickens (Figure 6). Based on a whole-genome study, the NL and NW lines both exhibit low genetic similarity with the commercial chicken, although they may be similar in terms of sharing selective sweep regions. Therefore, we presume that traits associated with the KNC NL and NW lines may be useful for improving egg and meat production, respectively, whereas the traits of other lines may be useful for improving both egg and meat production.

QTL regions specific to a chicken type were confirmed based on selective sweep regions identified through iHS and Rsb analyses of the commercial population (Table S4). QTL regions identified through iHS analysis in the layer population could be used to improve spawning ability, because they are associated with a small yellow follicle number, the FCR, ovary weight, and egg number. In the broiler population, selection signals were detected for QTL regions associated with growth properties (e.g., the FCR, breast muscle pH, and feed intake) and eggshell traits (e.g., eggshell strength and thickness). Similar results were obtained from the Rsb analysis. Significant QTL regions in the layer population were associated with feed intake, the FCR, ovary weight, albumen height and age at sexual maturity, whereas significant QTL regions in the broiler population were associated with the FCR, body weight, feed intake, and mean daily gain. Selection signals detected in the commercial population were found in specific QTL regions according to the chicken type; generally, there was continuous selection pressure on traits related to the FCR. QTLs associated with the FCR was detected in the G. gallus Chr 6: 7.9–12.1-Mb region, and it would be beneficial to improve these traits regardless of the chicken breed. Traits related to the FCR exhibit moderate heritability, and the FCR is used as an indicator of the degree of genetic improvement [50]. The KNC population has been maintained for the purpose of preservation, and the accumulation of relevant data is needed to improve their economic traits. To ensure profitability, chicken phenotypes should be continuously measured and recorded, and trait information regarding genes with selection signals should be used to improve the commercial population.

## 5. Conclusions

This study, we identified specific selective sweep regions in KNCs through selection signal analysis with commercial layers and broilers. Most QTL regions identified as candidate selection signal regions were associated with reproductive organs, eggshell traits, immunity, and organ development. The *LPP*, *IGF11*, *LMNB2*, *ERBB4*, *GABRB2*, *NTM*, *APOO*, *PLOA1*, *CNTN1*, *NTSR1*, *DEF3*, *CELF1*, and *MEF2D* genes produced haplotype block patterns with high LD values in regions exhibiting selection signals. Hence, the candidate regions likely underwent selection during environmental adaptation, and the selected traits may be useful for optimising productivity and further environmental adaptation in KNCs. From comparisons of selection signals with commercial chicken populations, we identified major economic traits that could be used for the efficient improvement of KNCs. According to comparative analysis with the layer population, major traits that underwent selection, were related to the FCR, ovary weight, albumen height, and age at sexual maturity. In contrast, major traits identified via comparative analysis with the broiler population were related to the FCR, body weight, feed intake, and mean daily gain. NL and NW lines contained a considerable number of selective sweep regions related to broilers and layers, respectively. This study provides novel insights into traits that underwent selective sweep, which could be used to genetically improve the KNC population.

## Figures and Tables

**Table 1:** Candidate gene lists of identified Rsb selection regions for 5 lines of Korean native chickens.

Comparison of Population		Candidate Gene (Excluded Unknown Gene)	Genes in High LD Region	QTL Name in High LD Region
Layer	NG	*ADGRL2/AHR/BANP/CACYBP/CADPS2/CD200/CHTOP/CNOT2/DOT1L/DPF3/DYNC1I1/ELF1/EPHA4/FBXW11/GRM8/HDAC9/HGFAC/HPCAL1/KCTD1/LOC112532947/MOB2/MYO18B/NARS2/OTUD7B/PAWR/PDE3A/PDLIM4/PDZRN3/PEBP1/PHF14/PLXNB2/POLA1/PPHLN1/RCOR1/RHOV/RNF216/RUNX1/SDHAF3/SLC15A4/SLC4A5/SNX13/TAOK3/UMAD1/UPK1B/USP15/WNT3A/YAP1*	*UPK1B/IGSF11/DPF3/PPHLN1/PPHLN1/PRICKLE1*	Breast muscle pH
	NL	*AEBP2/ANAPC4/B3GAT1L/CAMK2G/CHAT/CNOT2/DDX10/DIAPH3/DSCAM/ELF1/GALNT7/GALNTL6/KCTD1/LSAMP/MAP4K4/MYBPC1/PDE3A/PDK3/PHF14/PLXNB2/POLA1/PTK2/QTRT2/RAP1B/RAPGEF5/SEPSECS/SLC36A4/SNX13/SRRM1/STIM2/TBC1D19/TDRD3/TMEM245/UPK1B/USP15/VWA8/WASL/WNT3A/ZMIZ1*	-	-
	NR	*A4GNT/AGR2/ANKMY2/ANKRD28/BARX1/C1QTNF4/CELF1/CLASP1/CNTN5/CYP24A1/DIRC2/EP400/GALNTL6/GPR83/GRM8/IL1RAPL1/KAT2B/MAP4K4/MEOX2/MSRA/PAXIP1/PLXNB2/RAPGEF5/RPAP1/RYK/SDHAF3/SNX13/SPON1/STIM2/STK25/SYT10/TBC1D5/TOP2B/TSPAN7/USP25/WNT7A*	-	-
	NW	*ADCY9/AIFM3/ASB4/C22H2ORF42/CHN1/DDIAS/DPF3/DYNC1I1/EED/ELF1/EXT1/FSHR/GEMIN8/GPR158/HYAL6/IL1RAPL1/INO80/KIAA1217/LOC101751443/MCM5/ME3/MIPEP/MIR6672/NARS2/NOX4/OTUD7B/PDK3/POLA1/PRCP/RBFOX1/RGS6/SAT1/SDHAF3/SIPA1L1/SOSTDC1/STK3/SV2B/SYT1/TOP2B/TSPAN33/TSPAN7/TYRO3/UBE3B/UMAD1/USP15/VRK2/WDYHV1/YAP1/ZHX1/ZHX2*	*SIPA1L1/SV2B/SDHAF3/NOX4*	Fear—tonic immobility duration, breast muscle pH, feather-crested head, pH of digestive tract contents, tibia length, dry matter intake, feed intake, albumen height
	NY	*A1CF/AIFM3/AMBRA1/ANKRD33B/APPBP2/BRIP1/C22H2ORF42/CAMK2G/CERS6/CHN1/CREB3L1/CRIM1/DNAJC5/FBXW11/GALNTL6/GPR78/HTRA3/IGF2BP3/IL1RAPL1/KCTD1/KLHL41/LHX1/LRP2/LYPLAL1/MCM5/NEK6/PDE3A/PRPF6/RHOV/RNASEH2B/SERPINE3/SLC15A4/STIM2/STK3/TMEM132B/TTC3/VRK2/WASL/WDYHV1/ZFX/ZHX2*	-	-
Broiler	NG	*ACSBG2/AGRN/BMT2/CABIN1/CASTOR2/CNOT2/CNTN1/COMMD1/CUX1/DTNA/FARP2/FBXO42/GNA11/GPR132/HIF1A/INTS4/KCNC2/LOC112533299/MAP3K20/MIR6680/MOB1A/MOB2/MXD1/MYH10/NTSR1/OGFR/OTUD7B/P2RY8/PIAS4/PTPRF/PTPRT/PTTG1IP/RHOB/S100A6/SLCO4A1/TACR1/ZAP70/ZNF277*	*CNTN1/NTSR1/MEF2D*	Breast muscle pH, feed conversion ratio, comb weight, wattles weight, ileum weight
	NL	*ADIPOQ/ANAPC10/AUTS2/CDK8/CELF1/CNOT2/EFL1/GAB1/GAP43/GRID1/IGSF11/IL6/KCNC2/LMNB2/LOC112533299/LSAMP/MARCH2/MPRIP/MTFR1L/MYH10/PROM1/PTK2/PTP4A3/PTTG1IP/RUNX3/S1PR4/SLC10A7/SLC38A1/SNCG/SPON1/TDRD3/TTC32*	*MEF2D/LMNB2/LONP1/SLC1A6/RANBP3/KLHL33/NDUFA11/BTN1A1*	Feed conversion ratio, cecal bacterial burden after challenge with Salmonella T, ileum weight, comb weight, wattles weight
	NR	*A4GNT/ADD2/ALG10/ALKBH8/BICD1/C23H1ORF94/CABIN1/CELF1/CELSR1/CHMP4B/CPNE8/DMD/DVL1/FARP2/HDGFL1/IGSF3/IKZF2/LOC396098/LOC419409/MIR6680/MYH10/PTPRN2/PTTG1IP/RARB/SEMA5B/SINHCAF/SPDYA/TBC1D22A/TMTC1/TNRC6C/TOP1/TSPAN7*	*HDGFL1/RRNAD1/CRABP2/LOC425431/BCAN/HAPLN2/RHBG/MEF2D*	Feed conversion ratio, cecal bacterial burden after challenge with Salmonella T, ileum weight, comb weight, wattles weight
	NW	*CABIN1/CHD2/CNOT2/DMD/EIF2S3/ERBB4/GABRB2/HNRNPDL/INTS4/JMJD1C/KCNC2/LOC101751443/LOC112533299/MIR6672/MYO7A/NTM/OFD1/OTUD7B/PAK1/PDK3/POLA1/PRKCD/SLC38A1/SPON1/ZAP70*	*EIF2S3/APOO/MEF2D/POLA1/ERBB4/GABRB2/NTM/MIR1601*	Egg number, ovary weight, age at first egg, fear—tonic immobility duration, feed conversion ratio, feather pecking, testes weight
	NY	*CDC42BPA/CEBPG/CHMP4B/COX10/DSCAM/EPHB2/FAM18B1/FGFRL1/IKBKE/JMJD1C/KCNJ9/KIF16B/MRGBP/MYH10/PROM1/PURG/RAB18L/RIC3/SNRPB2/SSH2/STX8/ULK2*	*MEF2D*	Feed conversion ratio, ileum weight, comb weight, wattles weight

**Table 2:** Candidate gene lists of identical iHS selection regions for commercial and 5 lines of Korean native chickens.

Comparison of Population		Chromosome	Candidate Gene	QTL Name in Shared Selective Sweep Region
Layer	NG	8, 9, 19	*ACSL3/DTX2/ENSGALG00000045942/ENSGALG00000037501/SSC4D/ENSGALG00000017358/ENSGALG00000001926/SRRM3/MDH2/TMEM120A/ENSGALG00000002053/TAF15*	Feathered feet, wattles weight, fecal egg count, feed intake, feather pecking, alternative complement activation by BRBC, feed conversion ratio
	NL	3, 6, 11, 12, 14, 15	*CUZD1/C10orf88/SFMBT1/LMF1/ENSGALG00000006648*	Abdominal fat percentage, breast muscle weight, age at sexual maturity, ovary weight, ovary percentage, breast muscle pH, feed intake, dry matter intake, jejunum weight, feathered feet, wattles length, body weight (36 days), body weight, ileum length, body temperature
	NR	8, 11, 18	*ENSGALG00000005377*	Feathered feet, breast muscle pH, dry matter intake, jejunum weight, feed intake, body weight (hatch)
	NW	-	-	-
	NY	2, 11, 14, 21, 22, 28	*ADGRB1/ENSGALG00000032326/LMF1/KIF1B/ENSGALG00000002797/ENSGALG00000024481/DFFA/PEX14/DOT1L*	Wattles weight, breast muscle pH, feed intake, dry matter intake, jejunum weight, wattles length, body weight (36 days), body weight, gizzard weight, antibody titer to SRBC antigen, abdominal fat weight, breast muscle percentage, feather-crested head, gizzard percentage, eggshell color, small yellow follicle number, ovary weight, body weight (hatch)
Broiler	NG	7, 14, 24, 25	*SESTD1/ENSGALG00000004398*	Ovary weight, comb weight, body temperature, wattles length, breast muscle pH, fear—tonic immobility duration, feed conversion ratio, wattles weight
	NL	-	-	-
	NR	5	*RASGRP1/FAM98B*	-
	NW	1, 7, 14, 18, 24, 25	*ENSGALG00000001183/ENSGALG00000028706/ENSGALG00000024141/ENSGALG00000039987/ENSGALG00000028843/ENSGALG00000040425/ENSGALG00000045923/ENSGALG00000045397/ENSGALG00000037114/ENSGALG00000030308*	Breast muscle pH, ovary weight, comb weight, body weight (56 days), shank length, body temperature, wattles length, ileum weight, fear—tonic immobility duration, feed conversion ratio, wattles weight
	NY	7, 10, 14, 27	*ENSGALG00000033329/ENSGALG00000007687/ENSGALG00000004398/WNT9B/ENSGALG00000001079/ENSGALG00000001055*	Ovary weight, comb weight, body temperature, wattles length, wattles weight, pH of digestive tract contents, egg production rate

## Data Availability

The datasets analyzed in this study are available upon request from corresponding authors.

## Supplementary Materials

The following are available online at https://www.mdpi.com/article/10.3390/genes12060824/s1; Figure S1: result of admixture analysis cross-validation error plot, Figure S2: linkage disequilibrium block heatmap of myocardial development-related genes in commercial and Korean native chickens, Figure S3: linkage disequilibrium block heatmap of immune-related genes in commercial and Korean native chickens, Figure S4: linkage disequilibrium block heatmap of fear/behavior responses-related genes in commercial and Korean native chickens, Figure S5: linkage disequilibrium block heatmap of reproductive organ-related genes in commercial and Korean native chickens, Table S1: the results of candidate gene and QTL information contained in the selective sweep region identified through integrated haplotype homozygosity score (iHS) selection signal analysis in the Korean native chicken, Table S2: the results of candidate gene and QTL information contained in the selective sweep region identified through extended haplotype homozygosity ratio between populations comparative selection signal analysis (Rsb) between broiler and Korean native chicken, Table S3: the results of candidate gene and QTL information contained in the selective sweep region identified through extended haplotype homozygosity ratio between populations comparative selection signal analysis (Rsb) between layer and Korean native chicken, Table S4: the results of candidate gene and QTL information contained in the selective sweep region identified through integrated haplotype homozygosity score (iHS) and extended haplotype homozygosity ratio between populations comparative selection signal analysis (Rsb) between broiler and layer chicken.
