# Supplementary material for: Comparison of Selection Signatures between Korean Native and Commercial Chickens Using 600K SNP Array Data
Source: Genes (Basel). 2021 May 27;12(6):824. doi: 10.3390/genes12060824 (PMC8230197; doi:10.3390/genes12060824)
Supplement: Supplementary file 1 [file genes-12-00824-s001.zip › Figure S1-5.pdf]

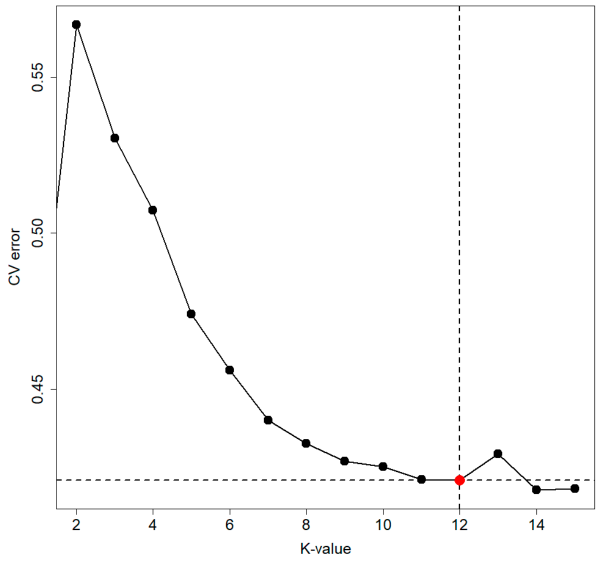

Figure S1. Result of admixture analysis cross-validation error plot.

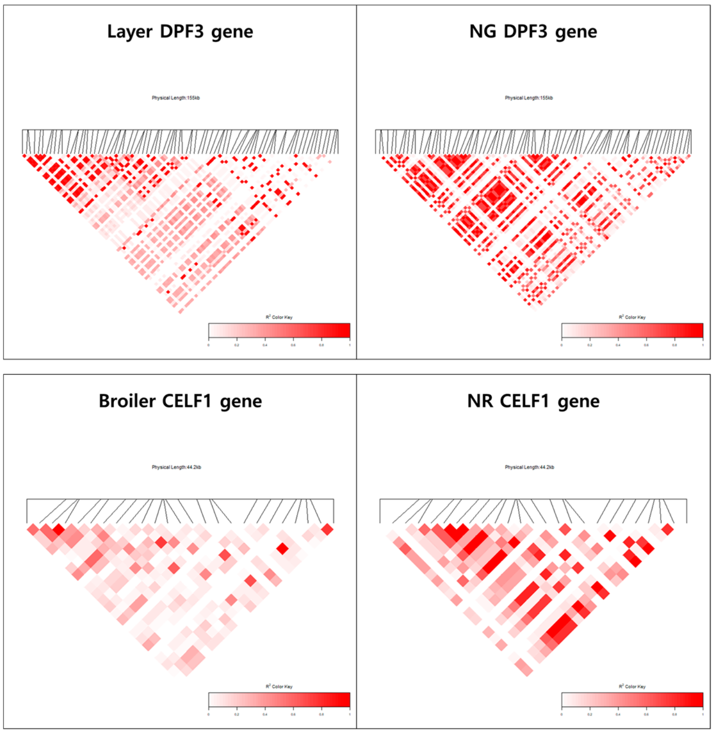

Figure S2. Linkage disequilibrium block heatmap of Myocardial development-related genes in commercial and Korean native chickens.

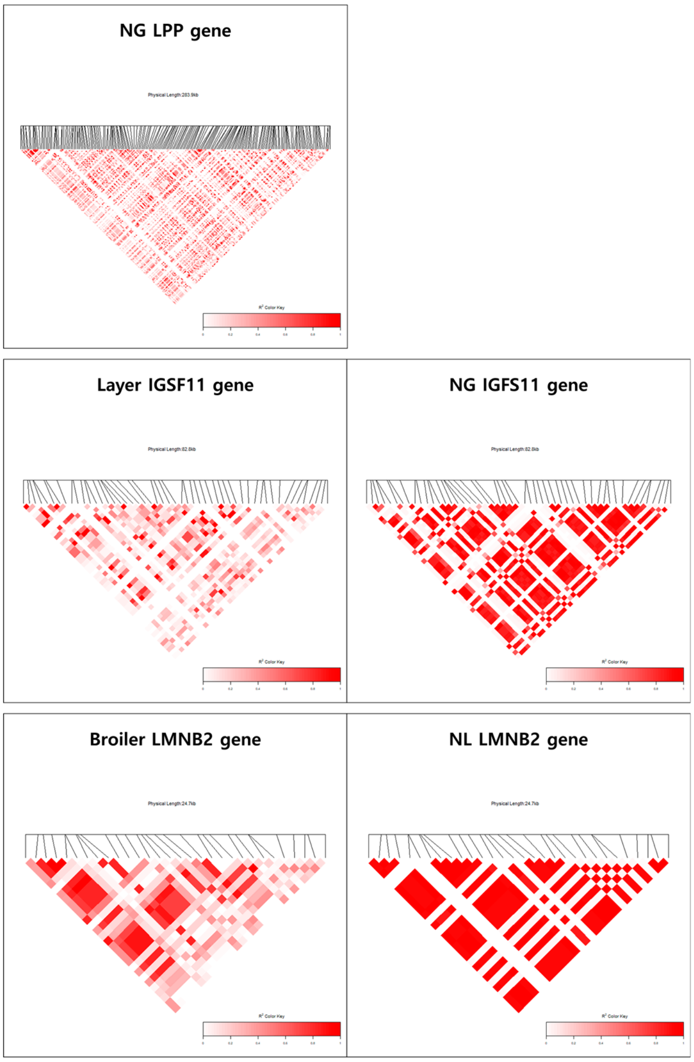

Figure S3. Linkage disequilibrium block heatmap of immune-related genes in commercial and Korean native chickens.

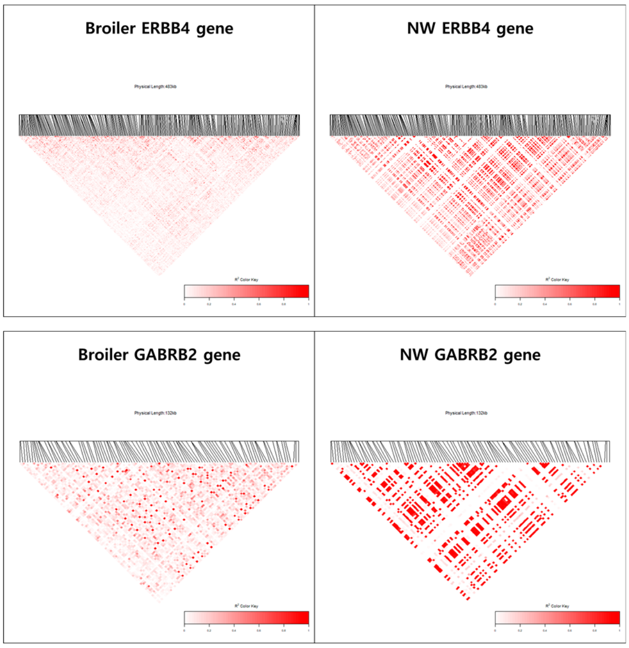

Figure S4. Linkage disequilibrium block heatmap of fear/behavior responses-related genes in commercial and Korean native chickens.

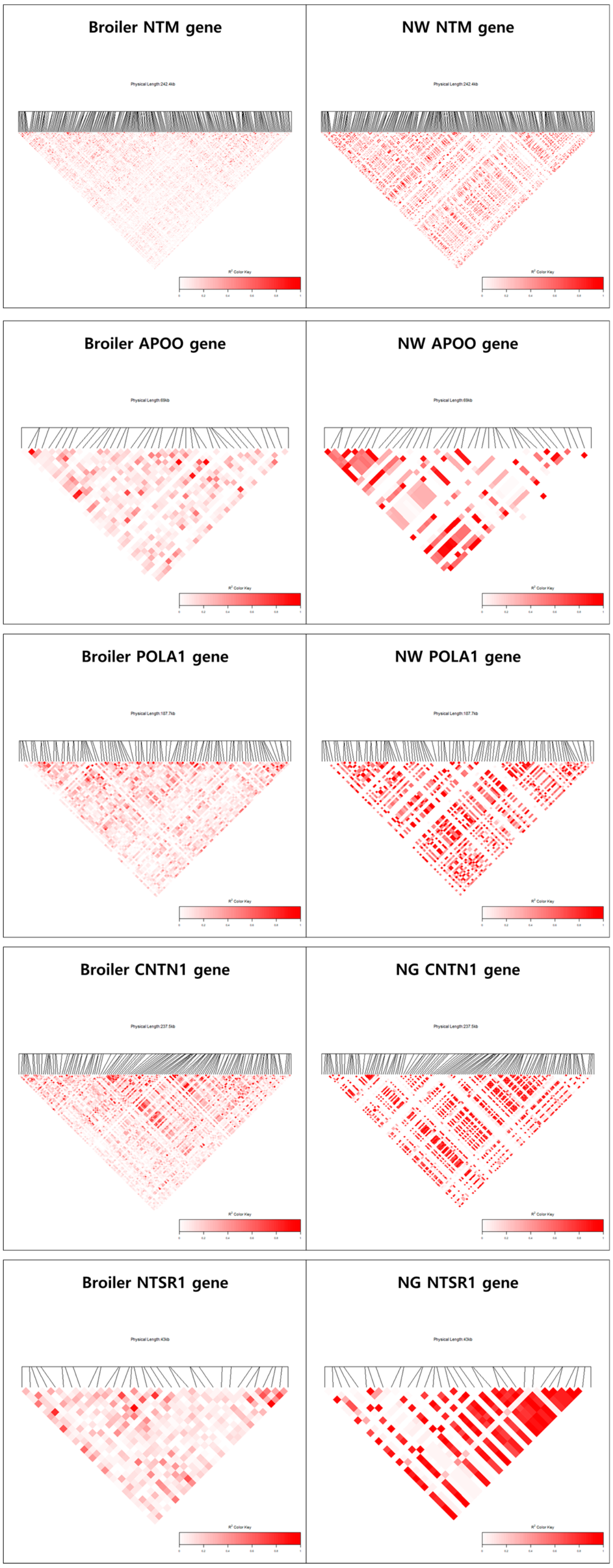

Figure S5. Linkage disequilibrium block heatmap of Reproductive organs -related genes in commercial and Korean native chickens.
